# A Method Enabling Comprehensive Isolation of Arabidopsis Mutants Exhibiting Unusual Root Mechanical Behavior

**DOI:** 10.3389/fpls.2021.646404

**Published:** 2021-03-03

**Authors:** Hiroshi Tojo, Aki Nakamura, Ali Ferjani, Yusuke Kazama, Tomoko Abe, Hidetoshi Iida

**Affiliations:** ^1^Department of Biology, Tokyo Gakugei University, Koganei, Japan; ^2^Nishina Center for Accelerator-Based Science, RIKEN, Saitama, Japan

**Keywords:** *Arabidopsis thaliana*, mechanosensing, mutant screening, method, mutagenesis, heavy-ion-beam irradiation, root behavior, sensor

## Abstract

Root penetration into soils is fundamental for land plants to support their own aboveground parts and forage water and nutrients. To elucidate the molecular mechanisms underlying root mechanical penetration, mutants defective in this behavior need to be comprehensively isolated; however, established methods are currently scarce. We herein report a method to screen for these mutants of *Arabidopsis thaliana* and present their phenotypes. We isolated five mutants using this method, tentatively named *creep1* to *creep5*, the primary roots of which crept over the surface of horizontal hard medium that hampered penetration by the primary root of the wild type, thereby forcing it to spring up on the surface and die. By examining root skewing, which is induced by a touch stimulation that is generated as the primary roots grow along a vertical impenetrable surface, the five *creep* mutants were subdivided into three groups, namely mutants with the primary root skewing leftward, those skewing rightward, and that growing dispersedly. While the majority of wild type primary roots skewed slightly leftward, nearly half of the primary roots of *creep1* and *creep5* skewed rightward as viewed from above. The primary roots of *creep4* displayed scattered growth, while those of *creep2* and *creep3* showed a similar phenotype to the wild type primary roots. These results demonstrate the potential of the method developed herein to isolate various mutants that will be useful for investigating root mechanical behavior regulation not only in Arabidopsis, but also in major crops with economical value.

## Introduction

Plant roots have a dual mechanical function as a sensor and drill. As a mechanical sensor, roots sense soil hardness, obstacles, and the gravity vector. As a drill, they thrust themselves deep into soils. A number of approaches have been employed to elucidate the molecular mechanisms regulating the mechanical features of the root. One effective approach is to screen mutants with altered root behavior and morphology. Isolated mutants include roots exhibiting a wavy growth pattern (Okada and Shimura, 1990), short root phenotype (Benfey et al., 1993), aberrant lateral root formation (Celenza et al., 1995), altered gravitropism (Bullen et al., 1990), and unusual plant hormonal responses (Maher and Martindale, 1980; Hobbie and Estelle, 1995). Consequently, many genes involved in primary root development have been identified and their products and roles have been characterized (reviewed in Oliva and Dunand, 2007; Monshausen and Haswell, 2013; Mai et al., 2014; Buschmann and Borchers, 2019; Su et al., 2020). These studies demonstrated that root mechanical functions are dependent on divergent cellular components, most of which are cytoskeletal proteins, receptor-like protein kinases, and auxin carriers. These characterized components appear to be involved in mechanotransduction and mechanoresponses rather than mechanosensing. Therefore, mutants with defective root mechanical functions have not yet been fully isolated and characterized. To cover such deficiency, an original methodology was needed.

In the present study, we developed a novel methodology to isolate these mutants in *Arabidopsis thaliana* (Arabidopsis, hereafter) based on the behavior of the roots of seedlings grown on impenetrable hard growth media. This screening method is expected to be easy to perform because the mutants to be screened keep growing under simple screening conditions that involve conventional media containing relatively high concentrations of gelling agents, such as agar and gellan gum. Here, we introduce the procedures employed in this screening method and demonstrate its usefulness as well as its potentially wide applications beyond model organisms and basic science.

## Materials and Methods

### Plant Materials

The *Arabidopsis* ecotype Columbia-0 (Col-0) was used in the present study as the wild type. The *axr4-2* mutant (Col-0 background; germplasm name CS8019), which was used as a control sample of root behavior, was obtained from the Arabidopsis Biological Resource Center (ABRC), Ohio State University, Columbus, OH, United States.

### Growth Conditions

Seeds were mixed for 10 min with 1 ml of 5% sodium hypochlorite/10% Triton X-100 solution, washed five times with autoclaved Milli-Q water (1 ml each time), and stored at 4°C for 3 days in the dark. Seeds were sown on 1/2 Murashige and Skoog (MS) medium (Murashige and Skoog, 1962; FUJIFILM Wako Pure Chemical Corp., Osaka, Japan) containing 0.8% agar (Difco Laboratories, Detroit, MI, United States), and germinated seedlings were grown under a 16 h-light/8 h-dark cycle with white light from fluorescent lamps at an approximately 40–60 μM m^−2^ s^−1^ light intensity at 22°C.

To specifically examine the morphology of the shoots and primary roots of the *creep* mutants, the seeds were sterilized and chilled as above and sown on 1/2 MS medium containing 0.4% (w/v) gellan gum (Nacalai Tesque, Kyoto, Japan), which is more transparent than agar, in rectangular plates (10 × 14 cm; Eiken Chemical Co., Ltd., Tokyo, Japan) to examine the shoots and custom-made growth-chambers made of glass (12 cm high × 14.5 cm wide × 2 cm deep in outer sizes) to examine the primary roots. Seedlings were grown under the conditions described above. The top view of shoots and the side view of primary roots were photographed 8 and 7 days after sowing, respectively.

### Irradiation of Seeds With ^12^C^6+^ Ions

The methods and conditions described by Kazama et al. (2011) were followed for the irradiation of seeds and growth of subsequent M_1_ seeds. Dry seeds of Arabidopsis (Col-0) were irradiated with ^12^C^6+^ ions with linear energy transfer (LET) of 30 keV/μm at a dose of 400 Gy using the E5 beam line at the RIKEN RI-beam factory as described previously (Kazama et al., 2011). M_2_ seeds obtained from 11 M_1_ plants were gathered in each pool, and 100 pools were sown for the first screening, as described below.

### First Screening of Mutants

A total of 200 M_2_ seeds were sown on 1/2 MS medium containing 5% gelrite (FUJIFILM Wako Pure Chemical Corp.) made in a rectangular plate (10 × 14 cm; Eiken Chemical Co., Ltd., Tokyo, Japan) and incubated for 10 days in the plate, which was placed horizontally, under the conditions described above. Surviving plants, which had true leaves and did not undergo necrosis, were selected and transferred to conventional 1/2 MS medium containing 0.8% (w/v) agar and then to the Arasystem 360 kit (Betatech BVBA, Ghent, Belgium) containing a 1:1 mixture of vermiculite (Fukushima Vermi, Fukushima, Japan) and Metro-Mix 350 (Sun Gro Horticulture, Agawam, MA, United States). Hyponex diluted 2,000-fold (N:P:K = 6.5:6:19) was added occasionally to the mixture as a fertilizer.

### Second Screening of Mutants

M_3_ seed aliquots (50 grains each) from two candidate mutants were sown separately with wild type seeds (20 grains) on the same medium as that used for the first screening and then grown for 15 days, after which the viability of seedlings was assessed for all candidates. Viability was examined in three independent experiments for each candidate mutant line. Viability was defined as the number of surviving seedlings vs. the total number of germinated seeds. Candidates with ≥38% viability were finally selected as genuine mutants.

### Skewing Experiments

Seeds of the mutants were sown on 1/2 MS medium containing 1.6% (w/v) agar. Plates were placed vertically, incubated for 7 days under the conditions described above, and photographed from the surface side of the medium. The skewed angle was assessed using ImageJ software (version 1.49v).

## Results

### Background of Mutant Screening

**Figure 1:**
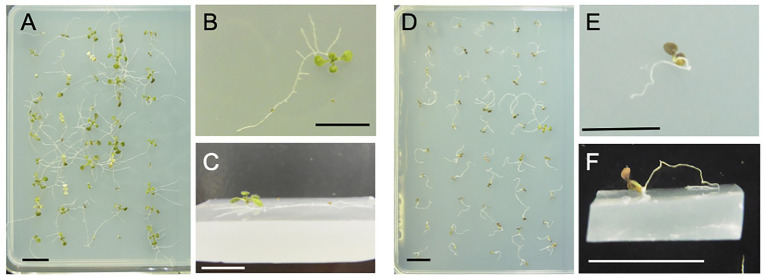
Phenotypes of two control lines used for screening in the present study. **(A–C)** The *axr4* mutant line and **(D–F)** the wild type (Col-0) line. Both lines were grown for 15 days on the surface of horizontal 5% gelrite-1/2 Murashige and Skoog (MS) medium. The primary and lateral roots of the *axr4* mutant crept over the surface of the medium and remained viable, while the primary root of the wild type sprang up on the surface, leading the seedling to die. The cotyledon and true leaves of the *axr4* mutant were green, while those of the wild type were brown, suggesting that the wild type underwent necrosis. **(A,D)** Above view; **(B,E)** close-up view from above; and **(C,F)** oblique close-up view. Scale bar = 1 cm.

The wavy root growth pattern of the Arabidopsis mutant *rgr1* (*reduced root gravitropism 1*; At1g54990), which is allelic to *axr4* (*auxin resistant 4*), varies on the surface of growth medium depending upon its tilt angles (Mullen et al., 1998). We examined the root behavior of this mutant using horizontally placed, 5% gelrite (a trade name of gellan gum)-solidified medium, which was hard enough to prevent penetration by primary roots. The merits of using such artificial hard media are that they are quickly and easily prepared, highly reproducible among independent experiments, whereby root behavior is clearly visible and monitored. As shown in Figures 1A–C, the primary and lateral roots of the *axr4* mutant crept over the surface of the medium, and the seedlings survived with green cotyledons and true leaves at 15 days after sowing. On the contrary, the primary root of the wild type sprang up on the surface, and the seedlings died with brown cotyledons (Figures 1D–F). These observations suggested that the *axr4* mutant was able to survive because root-medium contact allowed the plant to acquire water and nutrients for growth, and prompted us to develop a novel screening methodology to isolate mutants with altered root mechanical behavior.

### Screening of Mutants That Survive on Horizontal Impenetrable Medium

**Figure 2:**
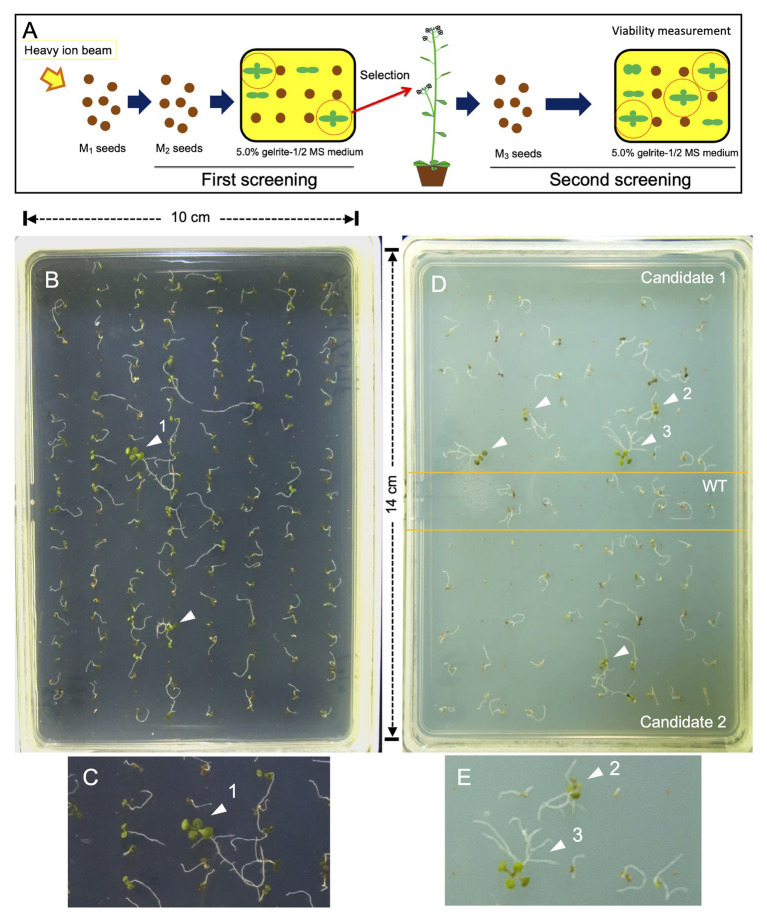
Screening method. **(A)** Schematic presentation of the flow of screening. **(B)** First screening. Two hundred M_2_ seeds were sown on 5% gelrite-1/2 MS medium, incubated for 10 days, and photographed. **(C)** Magnified image of seedling indicated by an arrowhead (number 1) shown in **(B)**. **(D)** Second screening. The M_3_ seeds (50 grains each) of two candidate mutants were sown separately with wild type (WT) seeds (20 grains), incubated for 15 days, and photographed. **(E)** Magnified image of two seedlings indicated by arrowheads (numbers 2 and 3) shown in **(D)**. The arrowheads in **(B–E)** indicate surviving seedlings that had green true leaves and did not undergo necrosis. Note that true leaves of seedlings 1 and 3 can be easily seen, while those of seedling 2 are less developed and hardly visible even after image magnification. Lateral roots are seen in seedlings 1–3, but not in those not pointed out by an arrowhead.

The method adopted for mutant isolation is shown as a schematic flow diagram in Figure 2A. Dry seeds of Arabidopsis (Col-0) were irradiated with ^12^C^6+^ ions as described previously (Kazama et al., 2011). One hundred pools of M_2_ seeds, each of which was obtained from 11 M_1_ plants, were sown for the first screening on the surface on 5.0% gelrite-containing 1/2 MS medium (Figure 2B). Ten days after seed sowing, the seedlings survived were transferred to conventional 0.8% agar-1/2 MS medium for further growth. Seeds harvested from self-pollinated plants were subjected to secondary screening with the same methodology to confirm their root phenotype (Figures 2B–E).

### Survival Rate of *creep* Mutants on Horizontal Impenetrable Medium

**Figure 3:**
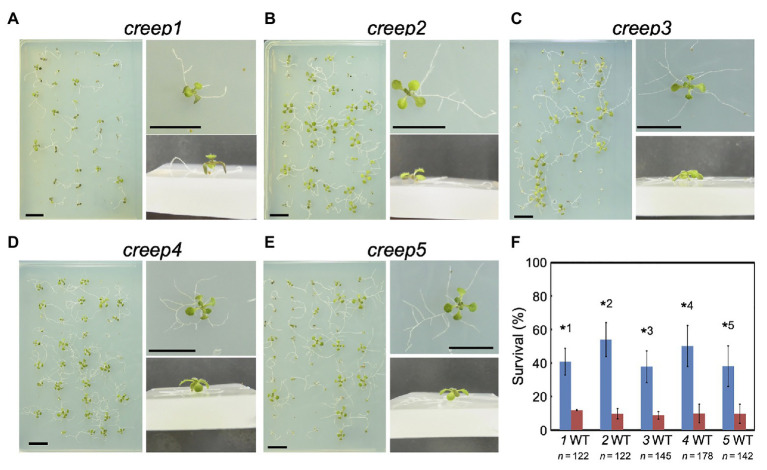
Phenotypes of *creep* mutants on horizontal impenetrable medium. The growth conditions used were the same as those described in the legend to Figure 1. Photographs were taken 15 days after sowing. **(A–E)** *creep1* to *creep 5* mutants. A set of three photographs (above view, close-up view from above, and oblique close-up view) are shown for each mutant. **(F)** Quantitative summary of viabilities assessed in at least three independent experiments. “*n*” represents the total number of surviving plants examined in three or more experiments. The numbers below the bar graph, 1–5, represent *creep1* to *creep5* mutants, respectively. The significance of differences between each mutant and the wild type was examined by the Student’s *t*-test. ^*^1, *p* = 0.0065; ^*^2, *p* = 0.0042; ^*^3, *p* = 0.0019; ^*^4, *p* = 0.0132; ^*^5, *p* = 0.0403. Significance was defined as *p* < 0.05.

**Figure 4:**
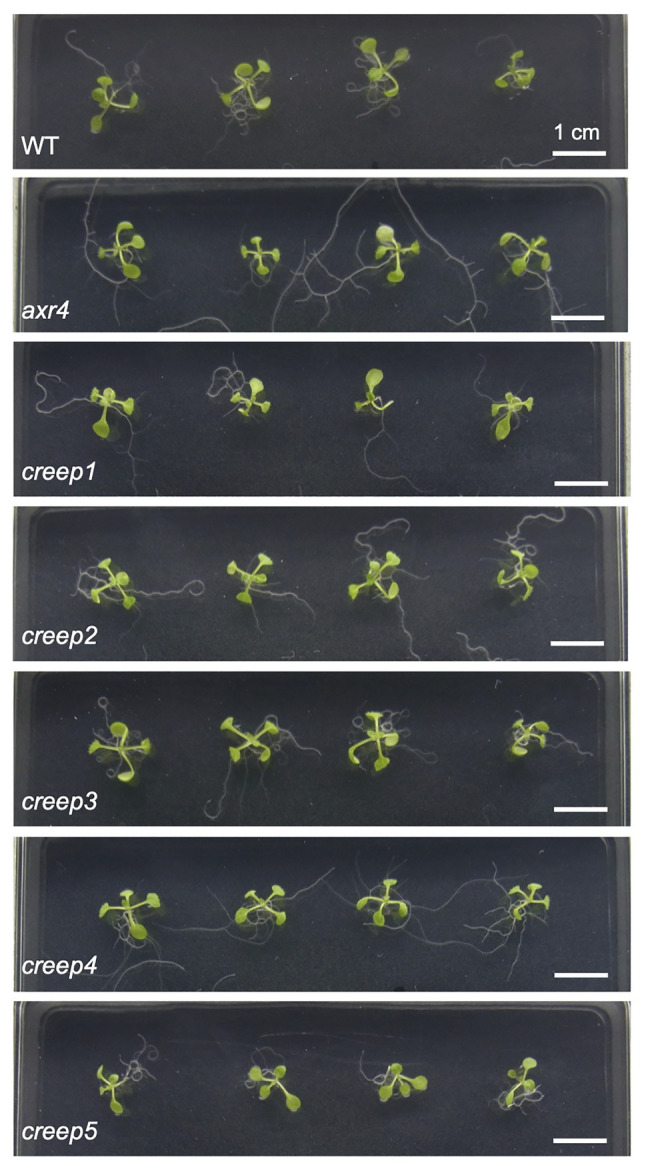
The top view of *creep* mutants on ordinary, root-penetrable 1/2 MS-0.4% gellan gum medium. The seeds of each line were sown and incubated at 22°C. Photographs were taken at 8 days after sowing. Scale bar = 1 cm.

**Figure 5:**
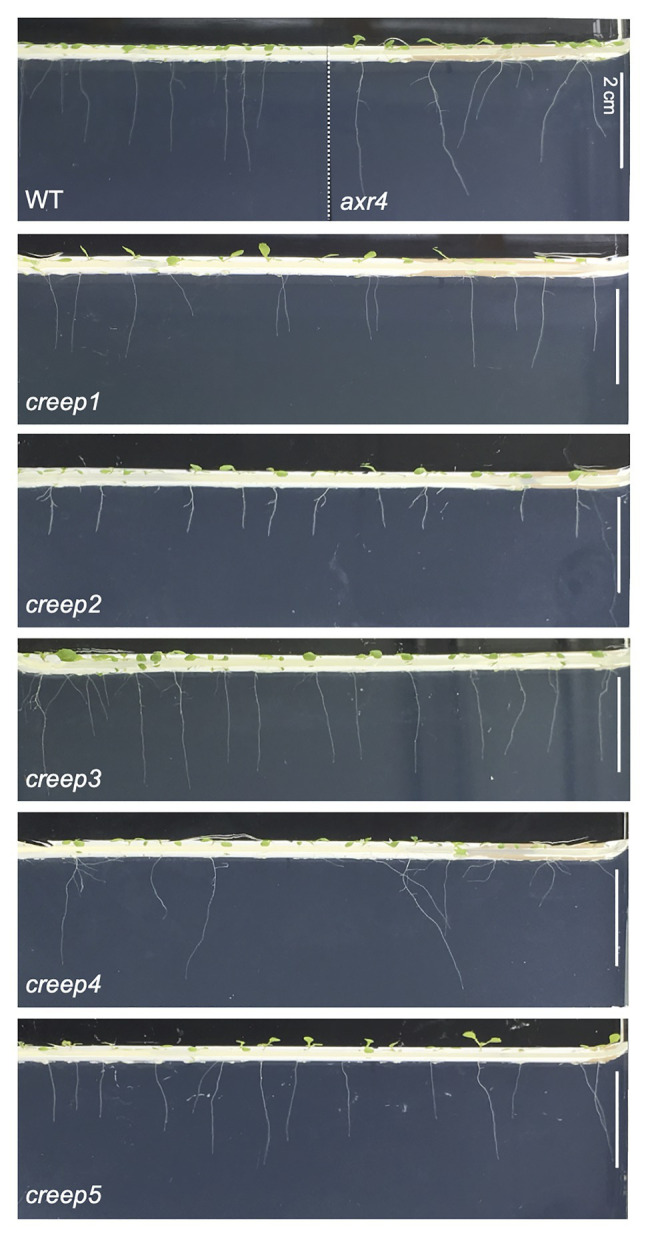
The side view of *creep* mutants on ordinary, root-penetrable 1/2 MS-0.4% gellan gum medium. The seeds of each line were sown and incubated at 22°C. Wild type and *axr4* plants were grown in the same growth chamber. Photographs were taken at 7 days after sowing. Scale bar = 2 cm.

The above procedure enabled us to isolate five mutants, the primary root of which crept over the surface of horizontal hard medium (Figures 3A–E). Note that each mutant was derived from a different M_2_ pool, and, therefore, we isolated five mutants from 1,100 M_1_ lines. We tentatively named these mutants *creep1–5*. A quantitative analysis revealed that the survival rate of the five mutants grown for 1 week on 5.0% gelrite-1/2 MS medium was approximately 30–60%, while that of the wild type was only 8–12% (Figure 3F). No significant differences were observed in the morphology or number of cotyledons and true leaves between the five *creep* mutants, the *axr4* mutant, and wild type plants grown on ordinary, root penetrable 0.4% gellan gum-1/2 MS medium when viewed from the top of the plates (Figure 4). On the other hand, the side view revealed some characteristic morphologies of the primary roots of the *creep2* and *creep4* mutants in the same medium: the primary root of the *creep2* mutant was shorter in length than the other mutants as well as wild type plants and that of the *creep4* mutant grew obliquely, reminiscent of the *axr4* mutant (Figure 5).

### Root Skewing Phenotypes of *creep* Mutants on Vertical Medium

**Figure 6:**
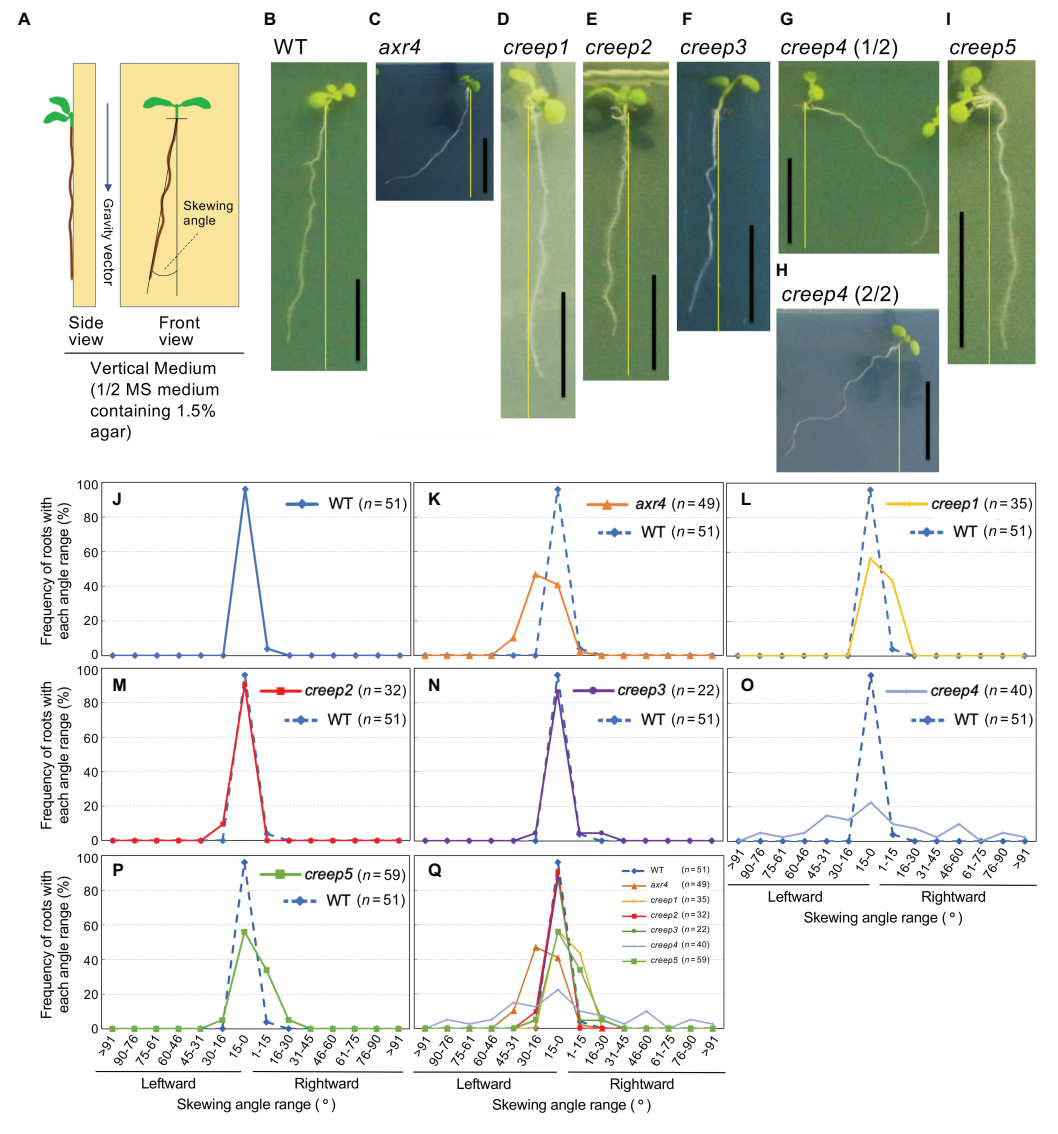
Root skewing phenotypes of *creep* mutants. **(A)** Illustration of vertical medium (left panel) and representation of how to measure the skewing angle (right panel). Skewing was quantified by measuring the angle between the vertical axis and root tip. **(B–I)** Photographs of the root skewing of *creep* mutants as well as the controls, wild type, and *axr4* mutants. The experiment was performed as described in the Materials and Methods section. Scale bar = 1 cm. **(J–P)** Quantification of the skewing angles of the *creep* mutants. According to the definition of the skewing angle shown in the right panel of **(A)**, the skewing angles of the *creep* mutants and the controls were measured, and the data of each *creep* mutant were individually compared to those of the wild type. Data of the wild type are shown with a dotted line in **(K–P)** for comparison. **(Q)** All the data in **(J–P)** are plotted together.

To characterize the root behavior of the *creep* mutants in more detail, we investigated their root skewing phenotypes on the surface of vertical agar medium according to a previously described method (Rutherford and Masson, 1996). The seeds of the mutants and control lines (the wild type and *axr4* lines) were sown on the surface of 1.6% agar-1/2 MS medium, grown vertically for 7 days (Figure 6A, left illustration), and photographed from the lid of the plates. The skewing angles of the primary roots of these samples were quantified using these photographs (Figure 6A, right illustration).

Figures 6B,J,Q show that the primary root of the wild type skewed slightly leftward (~90% 15–0° left), while that of *arx4* mutant was skewed strongly leftward (~45% 30–16° left and ~40% 15–0° left; Figures 6C,K,Q). The primary roots of the *creep1* and *creep5* mutants skewed slightly to either side (~55% 15–0° left and ~54% 1–15° right; Figures 6D,I,P,Q). Since the primary root of the wild type skewed leftward, the rightward skewing of the *creep1* and *creep5* mutants may be a unique phenotype of these mutants.

The primary roots of the *creep2* and *creep3* mutants skewed slightly leftward (~90% 15–0° left; Figures 6E,F,M,N,Q) at similar angles to the wild type. The primary root of the *creep4* mutant skewed dispersedly to either side (Figures 6G,H,O,Q).

## Discussion

In the present study, we developed a novel methodology to screen mutants with altered root behavior, which will potentially provide us with new mechanistic insights into root mechanosensing and mechanoresponses. Morphological responses to mechanical stress in plant roots have attracted the attention of many researchers and fueled lively work for decades, resulting in the discovery and characterization of various responses (for a review, see Potocka and Szymanowska-Pułka, 2018). We herein added a new morphological root response to mechanical stress generated by horizontally placed, impenetrable hard medium that does not allow roots to penetrate and forces wild type plants to die. Using this medium, we isolated five mutants, named *creep1*–*5*, which survived and grew on this medium through the creeping of their roots.

Based on the phenotypic variations of *creep* mutants (Figure 6), it was possible to predict the subclassification of the five *creep* mutations into at least three categories, namely, mutations resulting in leftward, rightward, and dispersed skews of the primary root. This prediction needs to be confirmed by the future genetic mapping and DNA sequencing of each mutated gene. It is important to note that the phenotypes of each of the five *creep* mutants in the present study are not necessarily caused by a single mutation. Nevertheless, these mutants clearly provide us with the opportunity to identify genes regulating the mechanical characteristics of the root, potentially resulting in novel mechanical insights. Furthermore, even if some *creep* mutants were produced by multiple mutations, they will contribute to the identification of functionally interdependent gene products.

We propose that the method described herein is useful for screening Arabidopsis mutants with a primary root capable of creeping on the hard, impenetrable surface of horizontal medium. We anticipate that this screening will contribute to the identification of gene products involved in mechanosensing that are poorly characterized as well as those required for mechanotransduction and mechanoresponses that have not yet been completely identified and characterized.

Since this method employs positive selection (i.e., the isolation of surviving mutants during screening), much more mutants may be isolated from Arabidopsis. This method would be applicable to major crop species, including rice, maize, and wheat, all of which have been used to investigate root-soil interactions and root responses to mechanical impedance (for reviews, see Kolb et al., 2017; Buschmann and Borchers, 2019).

## Data Availability Statement

The original contributions presented in the study are included in the article/supplementary material, further inquiries can be directed to the corresponding author.

## Author Contributions

HT performed the experiments and obtained the data presented in this manuscript. AN contributed to the initial stage of this research. AF supervised HT to perform some of the experiments. YK and TA contributed to the irradiation of seeds. HI conceived and designed the research and wrote the first manuscript draft. All authors reviewed the draft and approved the final version of the manuscript.

### Conflict of Interest

The authors declare that the research was conducted in the absence of any commercial or financial relationships that could be construed as a potential conflict of interest.
